# Mapping the knowledge of scientific production on the ALPHA-Fitness battery at school age: bibliometric analysis (2010–2025)

**DOI:** 10.3389/fpubh.2026.1885114

**Published:** 2026-10-13

**Authors:** Juan David Paucar-Uribe, Boryi A. Becerra-Patiño, Anelise Reis Gaya, Zhen Yang, Patricio Solis-Urra, Júlio Brugnara Mello, José Francisco López-Gil, José Castro-Piñero, Jorge Olivares-Arancibia

**Affiliations:** 1 Faculty of Physical Education, National Pedagogical University, Bogotá, Colombia; 2 Projeto Esporte Brasil (PROESP-Br), Universidade Federal de Pelotas, Pelotas, Brazil; 3 Department of Movement Sciences, KU Leuven, Leuven, Belgium; 4 Facultad de Educación y Ciencias Sociales, Universidad Andrés Bello, Viña del Mar, Chile; 5 AdventHealth Research Institute, Neuroscience Institute, Orlando, FL, United States; 6 Grupo de Investigación en Adaptaciones Musculoesqueléticas al Entrenamiento, Escuela de Educación Física, Pontificia Universidad Católica de Valparaíso, Valparaíso, Chile; 7 School of Medicine, Universidad Espíritu Santo, Samborondón, Ecuador; 8 Faculty of Health Sciences, Universidad Autónoma de Chile, Temuco, Chile; 9 GALENO Research Group, Department of Physical Education, Faculty of Education Sciences, University of Cadiz, Cádiz, Spain; 10 Instituto de Investigación e Innovación Biomédica de Cádiz (INiBICA), Cádiz, Spain; 11 AFySE Group, Research in Physical Activity and School Health, School of Physical Education, Faculty of Education, Universidad de las Américas, Santiago, Chile

**Keywords:** childhood, physical activity, physical fitness, preadolescence, sedentary behavior, bibliometric analysis, ALPHA-Fitness battery

## Abstract

**Introduction:**

Numerous studies have used the Assessing Levels of Physical Activity and Fitness (ALPHA) battery to evaluate physical fitness (PF) and its positive impact on the health of school-age children, assessing various components of fitness. Despite its widespread use, no bibliometric review has systematically examined research trends or scientific output related to the ALPHA-Fitness battery. This study aimed to conduct a bibliometric review to identify research trends on the ALPHA-Fitness battery.

**Materials and methods:**

Theoretical study using bibliometrics as a research technique, following the Preferred Reporting Items for Systematic Reviews and Meta-Analyses (PRISMA) methodology. A total of 266 documents published between January 1, 2010, and February 28, 2025, were identified in the Web of Science, Scopus, and PubMed databases and analyzed using VOSviewer software (v.1.6.19). The search was independently reviewed and verified by two researchers. The final search equation applied was (Alpha fitness test OR alpha fitness battery OR Alpha-fitness test OR fitness alpha test OR alpha fitness test battery AND physical fitness).

**Results:**

The final sample consisted of 266 documents, mostly published as research articles (98.87%). Scientific output was highest between 2019 and 2024 (*n* = 184), with 2019 also recording the most citations (*n* = 807), followed by 2017 (*n* = 767). López-Gil was the most prolific first author, with eight documents and 130 citations. The most frequent keywords were Children (*n* = 43), Adolescents (*n* = 34), Cardiorespiratory fitness (*n* = 28), Physical fitness (*n* = 21), Obesity (*n* = 20), and Childhood (*n* = 19). Spain led in publication output (63 articles, 2,130 citations), followed by Sweden (14 articles, 1,469 citations) and Chile (13 articles, 91 citations).

**Conclusion:**

The analysis of the existing literature on the ALPHA-Fitness battery reveals a predominance of studies focused on the battery itself (*n* = 210 documents), compared to studies involving the administration of at least one test from the battery (*n* = 56 documents).

## 1 Introduction

Physical fitness (PF) is widely recognized as a powerful and multidimensional health marker in children and adolescents (1). Robust evidence from systematic reviews and meta-analyses (2, 3) indicates that higher levels of PF are positively associated with cognitive functioning (4, 5), academic performance (6, 7), and mental health outcomes, including lower levels of depression and anxiety, as well as improved self-esteem and overall well-being (8). Conversely, low PF has been linked to negative health-related behaviors such as increased sedentary time and a higher likelihood of alcohol and tobacco use (9). From a physiological perspective, PF is also strongly associated with key cardiometabolic health indicators, including reduced risk of type 2 diabetes (10), hypertension (11), and obesity (12). Given this broad impact across cognitive, psychological, behavioral, and physical domains, PF can be considered an integrative marker of current and future health status. Therefore, the accurate assessment and monitoring of PF in youth populations is essential to identify risk profiles, guide interventions, and support health promotion strategies in school settings.

In this context, the assessment of PF is conducted using standardized and validated field-based tests that allow the monitoring of physiological adaptations with adequate reliability and reproducibility (13, 14). Health-related PF assessments commonly include body composition indicators (e.g., body mass index (BMI) and waist circumference), cardiorespiratory fitness (CRF), and muscular fitness components such as strength and power (2). Despite the availability of multiple testing batteries, previous evidence has identified up to 25 health-related PF batteries, highlighting CRF and muscle strength as core components across all protocols (15). Among these, the extended Assessing Levels of Physical Activity and Fitness (ALPHA) test battery has emerged as one of the most widely used and scientifically supported instruments for assessing health-related PF in children and adolescents (16, 17). According to Ruiz et al. (16), the ALPHA-Fitness battery was developed in three versions: (i) an evidence-based battery, (ii) a high-priority battery designed for time-efficient assessments, and (iii) an extended battery recommended for epidemiological studies. The extended version includes assessments of cardiorespiratory fitness (20-m shuttle run test), muscular strength (handgrip strength and standing long jump), motor fitness (4 × 10 m shuttle run test), and body composition (e.g., BMI and waist circumference), thereby providing a comprehensive evaluation of health-related PF in children and adolescents.

In addition, the ALPHA-Fitness battery has been extensively applied across different populations and research contexts, contributing to a growing body of evidence linking PF with cardiometabolic health, cognitive outcomes, and overall well-being. This widespread adoption, together with its standardized structure and adaptability through its different versions (i.e., evidence-based, high-priority, and extended), positions the ALPHA-Fitness battery as a reference tool for both research and applied settings.

Given its widespread use and growing importance, it is important to systematically examine how scientific knowledge regarding the ALPHA-Fitness battery has evolved over time. Understanding patterns of scientific output can provide valuable insights into leading countries, institutions, and authors, as well as major research trends and emerging topics within this field, in addition to identifying theoretical gaps and tracking the evolution of the subject matter. Despite the growing number of studies using this battery, no bibliometric analysis has yet been conducted that comprehensively maps the scientific output related to its application. Such an analysis is essential for identifying knowledge gaps, consolidating existing evidence, and guiding future lines of research. Therefore, the objective of this study was to analyze scientific output related to the use of the ALPHA-Fitness battery in children and adolescents using the VOSviewer and Bibliometrix software.

## 2 Materials and methods

### 2.1 Design

The present study used bibliometric as a research technique (18). Through the classification carried out by Passas (19), the present study was constituted as research of a theoretical nature because the main objective was to examine and outline a large amount of information associated with the number of publications made according to time and their evolution to the present (20, 21). This type of bibliometric analysis is based on different criteria to map knowledge on a particular topic from studying institutional affiliations, academic cooperation between researchers, publishing groups, countries, keywords, and cited references (22) through quantitative analysis.

### 2.2 Data extraction

The review of the articles began on February 28, 2025, and was analyzed and verified by two researchers using the databases. These databases have been used in numerous bibliometric analysis articles on various research topics related to sports science and physical activity (PA) (23–25). In accordance with the Preferred Reporting Items for Systematic Reviews and Meta-Analyses (PRISMA) guidelines, various search queries were formulated for the Web of Science (WoS), PubMed, and Scopus databases. Among these, the most relevant MeSH terms and keywords for the topic were considered: (“Alpha Fitness Test Battery” [All fields]) OR (“Alpha Fitness Test” [All fields]) OR (“Alpha Fitness” [All fields]). To this end, various search queries were developed using Boolean operators such as “OR” and “AND” without using “NOT,” since this operator excluded items that could be considered key to the present review. Once the search strategies were established, they were analyzed to ensure consistency and avoid biases that could lead to the exclusion of articles from the review. Accordingly, the results were reviewed, including the title and abstract-taking into account the number of articles found and the research topic; thus, the most appropriate search formula was ((Alpha fitness test OR alpha fitness battery OR Alpha-fitness test OR fitness alpha test OR alpha fitness test battery) AND physical fitness). The search was conducted on February 28, 2025, as reported in Table 1.

**Table 1:** Equation for the search of studies, date, number of articles and type of documents.

Boolean operators	Search date	Publication date intervals	Database	Total number of found articles
Alpha fitness test OR alpha fitness battery OR Alpha-fitness test OR fitness alpha test OR alpha fitness test battery AND physical fitness	February 28, 2025	2010–2025	Web of Science	1,089
			Scopus	145
			PubMed	539

* This formula is considered by the approximation of the study theme and number of articles found.

During the extraction and retrieval of the documents found in the search, 1,773 documents were identified in the databases and 121 documents were identified using other methods (Google Scholar and citation searching). The analysis was conducted and verified by two of the principal investigators (BB-P and JP). A systematic order was established based on the identification, selection, and inclusion phases to create the flowchart. Duplicate articles were discarded, and only those documents that addressed the research topic and met the inclusion criteria were considered (22). Figure 1 illustrates the identification, screening, and eligibility phases, through which the documents passed to compile the results; ultimately, a total of 266 documents were considered, all of which met the researcher’s eligibility criteria.

**Figure 1:**
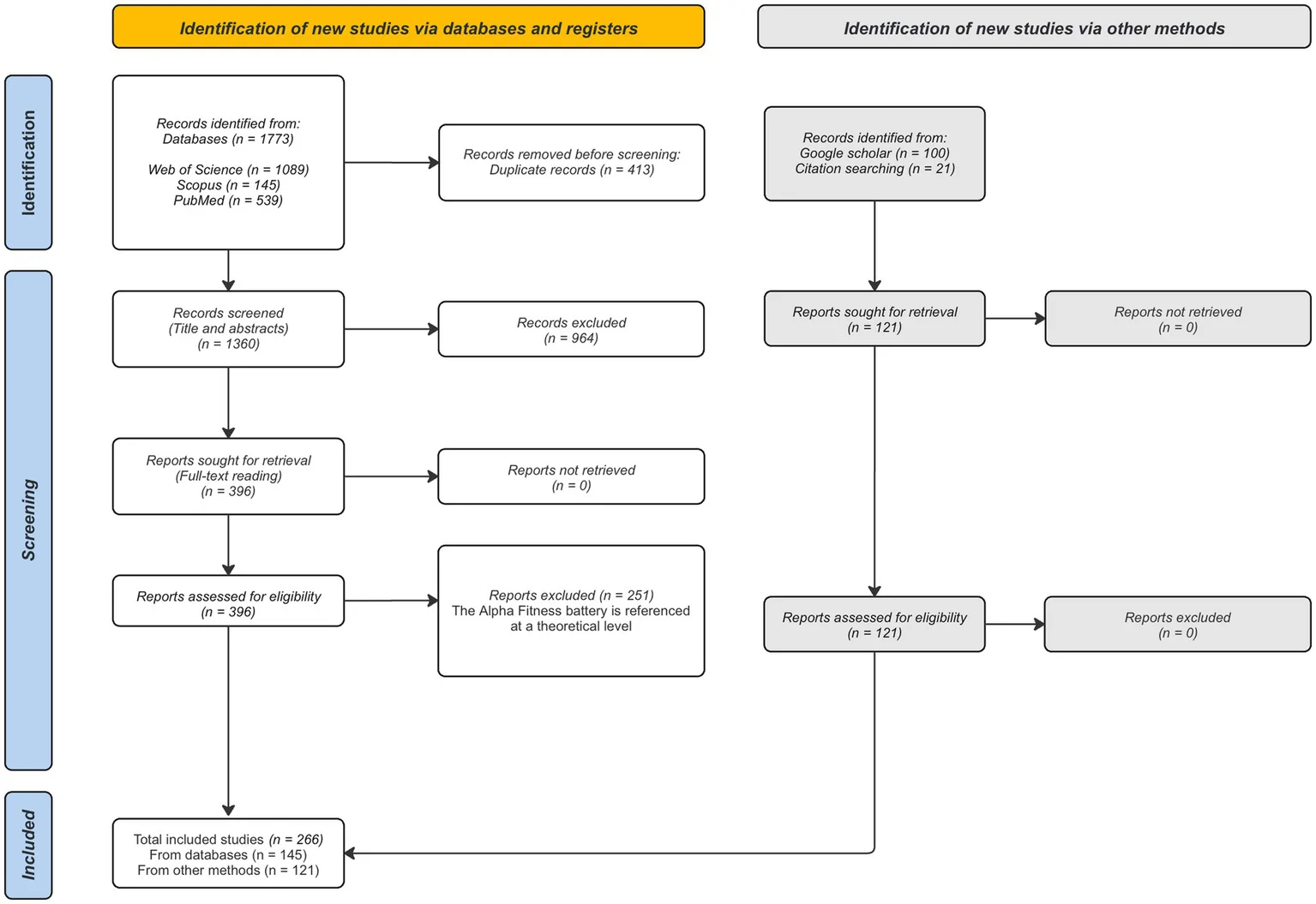
Flow chart for the selection of studies according to PRISMA guidelines.

Data extraction followed a standardized protocol. Each document retrieved from the databases was extracted, and each of the analysis variables contained in the article such as title, authors, year, DOI, and others were independently verified. The data was then standardized so that the software could interpret it without generating ambiguity. All documents were analyzed; any discrepancies found in a document were resolved by consensus. Once the documents were finally standardized and free of ambiguity, they were subjected to analysis.

### 2.3 Eligibility criteria

The selection criteria established by the researchers were the following: (i) documents published between January 1, 2010, and February 28, 2025; (ii) documents that were in their entirety and with free access; (iii) the subject of study should have a focus on schoolchildren and adolescents; (iv) documents published in English or Spanish; (v) research reviewed by academic peers and with original authorship; (vi) the classification of the articles will be in the quartiles Q1, Q2, Q3, and Q4 so that the publication journal was indexed and classified in Journal Citation Report (JCR) or Scimago Journal Rank (SJR).

### 2.4 Data analysis

The identified data were extracted in two different formats: plain text and Excel. The Excel document was used to perform a descriptive analysis of the results using a Microsoft Excel spreadsheet (v. 2010, Microsoft Corporation, Redmond, WA, United States). The data were analyzed by title, abstract, authors, year, and DOI to standardize the data and avoid bias in the findings and were subsequently stored and uploaded to the analysis software. The plain text file allows for analyses of co-authorship by authors, co-authorship by organizations, keyword occurrence, citations by document, citations by journal, citations by country, and citations by authors using the VOSviewer program (v. 1.6.19, Center for Science and Technology Studies, Netherlands). A fragmentation analysis was performed with an attraction value of 3 and a repulsion value of −3. The VOSviewer program allows for the creation of two-dimensional graphs (26) based on nodes to establish the main relationships that emerge from the scientific output in the documents indexed in the databases (27). The Bibliometrix program was also used, which provided additional relevant information for the study’s topic.

To substantiate the analysis carried out, several bibliometric laws were used: (1) Price’s law for literature growth; (2) the *R*^2^ of the time-series linear fit between the number of published documents (and citations) and year (28); (3) Lotka’s law allows identifying the authors who have published the most studies (29, 30); (4) Zipf’s law is oriented to establish the occurrence of the most used keywords (31). Likewise, the H-index was used, which determines academic productivity based on how a number *h* of documents has been cited at least a minimum *h* of times (32).

## 3 Results

### 3.1 Evolution of the number of documents

The publications of the ALPHA-Fitness Battery are observed for each of the years (Figure 2) between 2010 and 2024. It is observed that the years with the lowest number of publications were between 2010 and 2014. On the other hand, the years with the highest scientific production are established in the years 2019–2024, with a total of 184 published studies. The years of publication with average trends are 2017 and 2018. According to the trend line, there has been marked growth over the years. However, academic productivity between 2010 and 2018 was only 30.82%, and between 2019 and 2024, it represented 69.2%. Therefore, in the last 6 years, according to the *R*^2^ coefficient (*R*^2^ = 0.9242), its increase has generated a growing interest in the Sports Sciences community.

**Figure 2:**
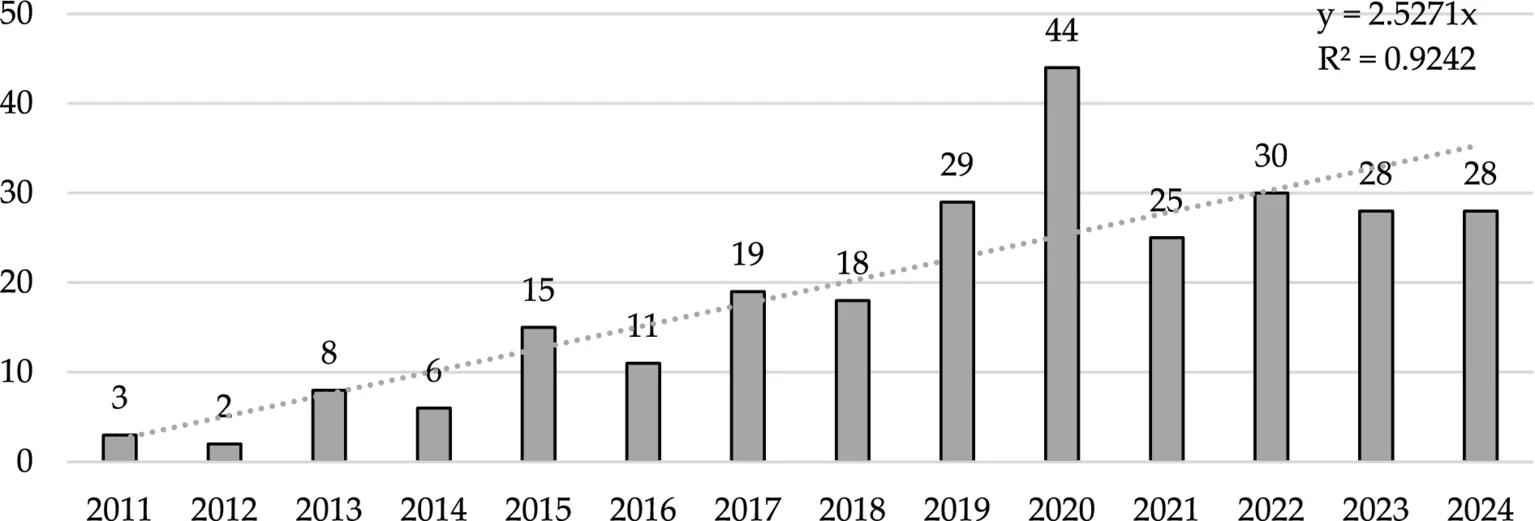
Evolution of the number of annual publications.

Figure 3 shows the number of citations per year, where the trend line is oscillatory. In 2019, the most significant volume of citations was presented (*n* = 807), followed by 2017 (*n* = 767). The lowest levels of citations were recorded in 2012, 2023 and 2024. Likewise, the years with the greatest drop in the number of citations were 2012 and 2021.

**Figure 3:**
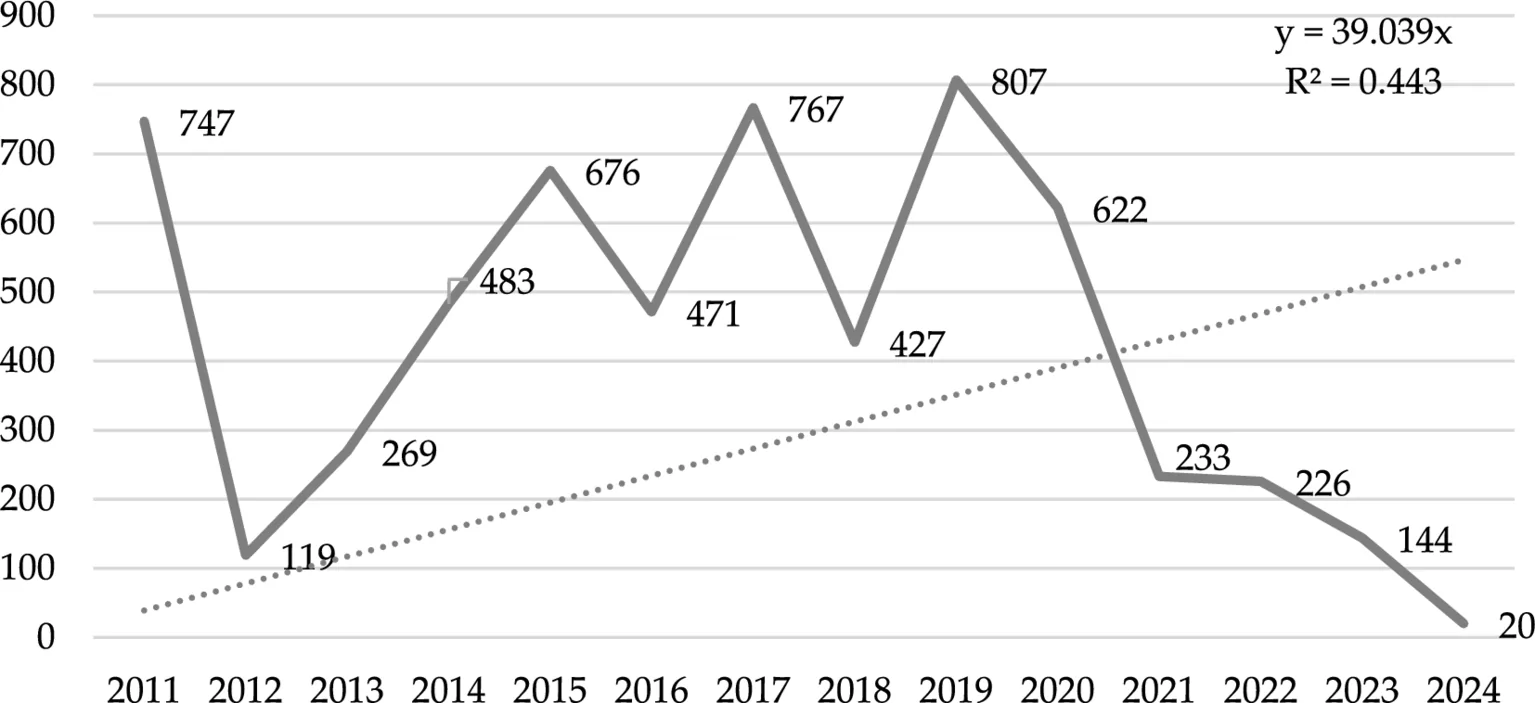
Evolution of the number of annual citations.

### 3.2 Documents by type

The authors have shown greater interest in conducting research articles using the ALPHA-Fitness battery, which results in 98.87% of the total being obtained in this model. Subsequently, the review meant 1.13%, the minimum value for the document type (see Table 2).

**Table 2:** Type of published documents.

Type document	Amount of documents	Percentage %
Original research article	263	98.87
Review	3	1.13
Total	266	100

### 3.3 ALPHA-Fitness battery type analysis

The evaluation of the physical condition of children and adolescents has allowed for constant modifications within the same battery. The high-priority test consists of aerobic capacity (20 meters test), Skeletal muscle capacity (handgrip strength and long jump), and body composition (body mass, BMI, waist circumference). Based on the evidence, the battery has the same characteristics, adding the skin folds; the extended test consists of the same variables, adding the motor capacity in the speed-agility test 4 × 10 meters (16). Finally, the total use of the ALPHA-Fitness battery in the studies represents more than two thirds (210/266; 78.9%) of the included studies. On the other hand, there are several studies that only use at least one ALPHA-Fitness battery test. The most commonly used ALPHA-Fitness battery is the 20-meter test. Thus, as of February 28, the study developed by Ruiz et al. (17) had received a total of 543 citations and the total citations achieved by the 266 included studies was 6,011 citations. Thus, 5,038 citations have cited the complete ALPHA-Fitness battery and 973 citations have been cited in articles that only used at least one test of the battery (see Table 3).

**Table 3:** ALPHA-Fitness battery type.

Battery type	Number of documents	Number of citations	Percentage % of citations
ALPHA-Fitness Battery	210	5,038	83.81
At least one battery test	56	973	16.19
Total	266	6,011	100

### 3.4 Trend topics

Upon analyzing the most relevant topics detailed in Figure 4, it is clear that, for the years 2019 and 2021, these were: human, male, fitness, and female. For the year 2019, an increase in the number of studies is evident for concepts such as: cross-sectional study, muscle strength, and female. For the year 2020, the concepts that reached at least 300 results were: human, male, and fitness. Finally, for the year 2023, the concepts that are being studied and stand out are: aerobic exercise, World Health Organization, and learning.

**Figure 4:**
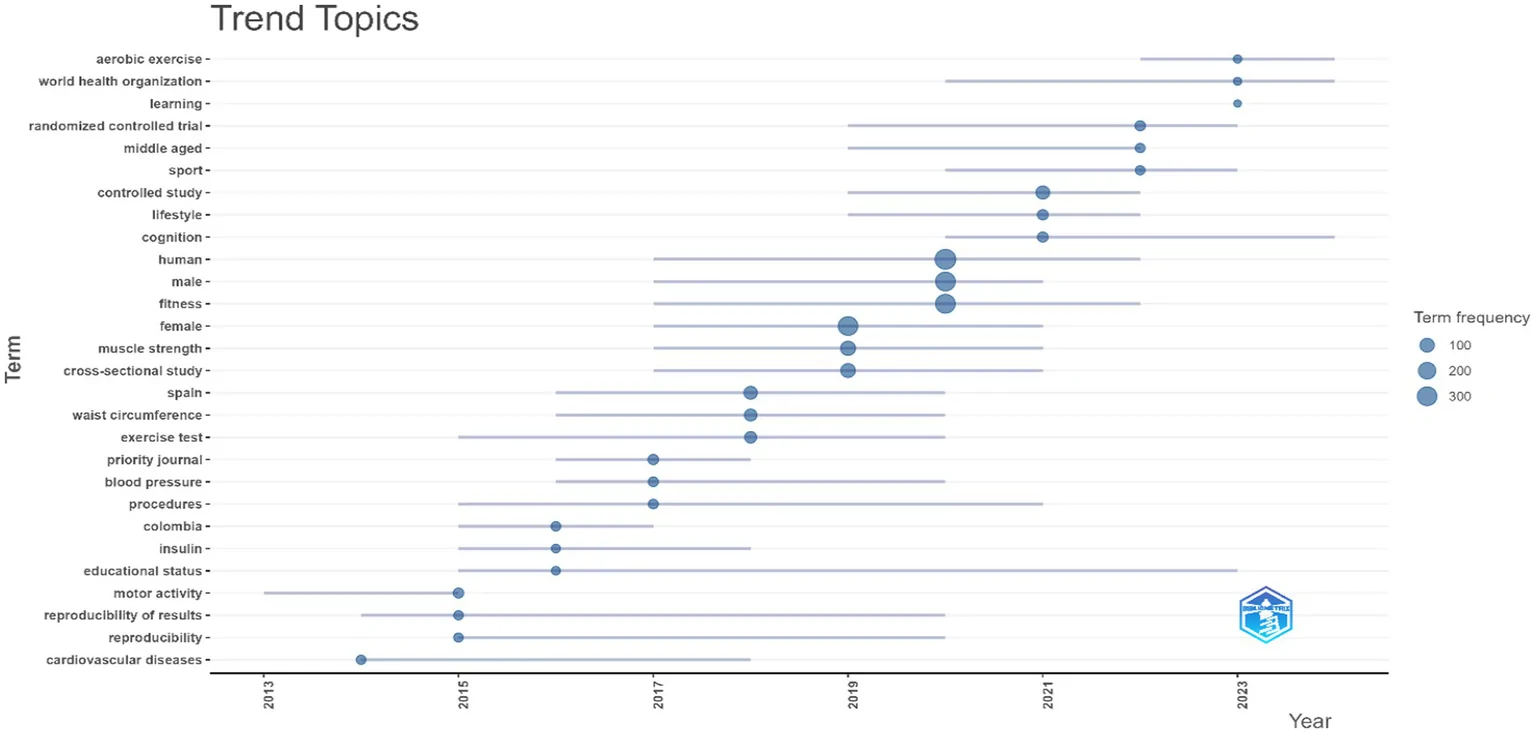
Trend themes. The X-axis represents the year and the Y-axis represents the cumulative occurrences in response to the number of keywords.

In addition, Figure 5 reveals the concepts used in the different categories. Thus, the specialized terms are related to nutrition: the Mediterranean diet and metabolic syndrome. The search topics are grouped under physical fitness, which is closely linked to education and mental health, with high centrality and evolution. Finally, the emerging or declining terms that appear are: adiposity and body composition, which help us understand the shift in scientific interest from focusing solely on body measurements to more multifactorial analyses. Another key finding involves basic terms such as “obesity,” “physical fitness,” and “cardiorespiratory fitness,” which exhibit a moderate level of centrality, although their dimensionality ranges from low to moderate, enabling the identification of future lines of research for ALPHA-Fitness.

**Figure 5:**
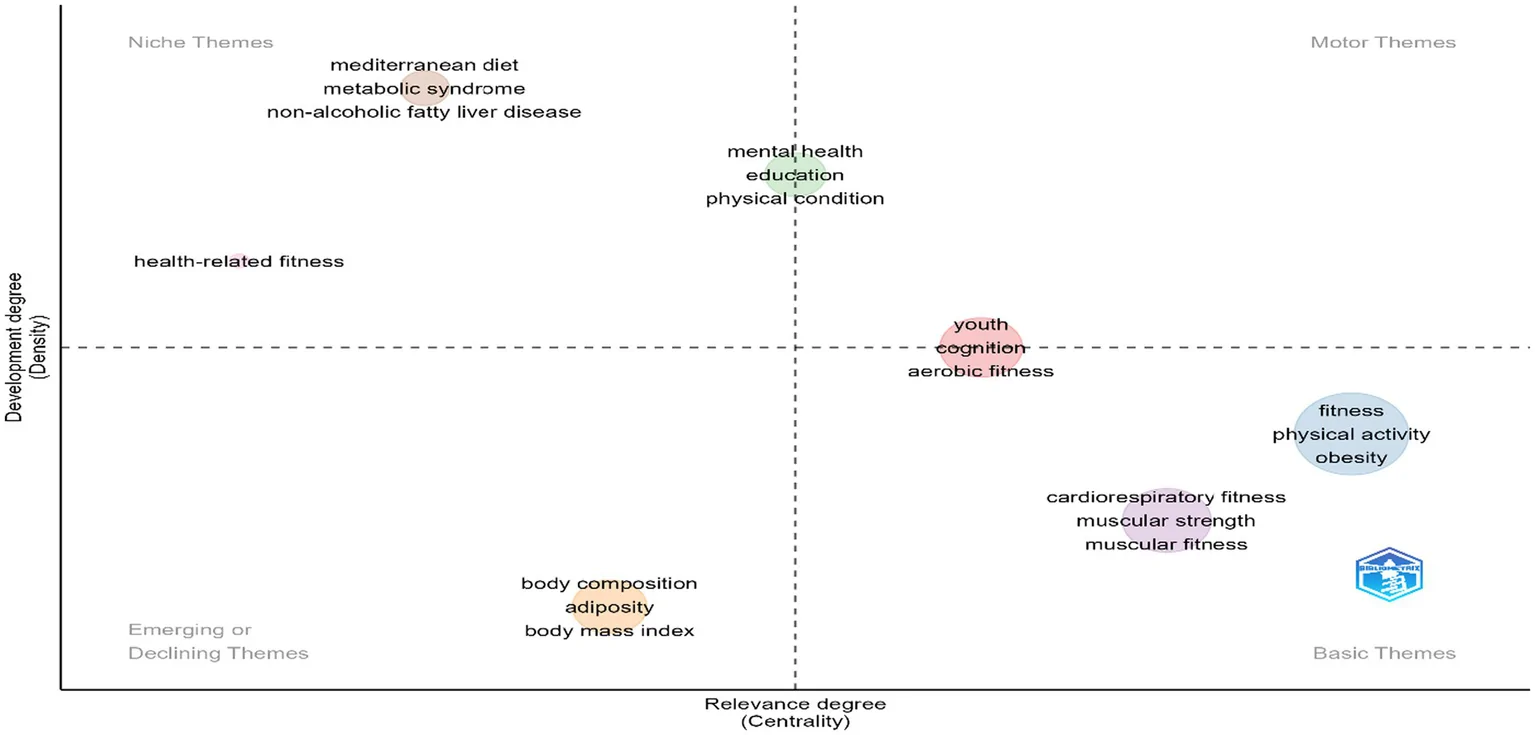
Degree of development and density of key concepts in the ALPHA-Fitness battery study.

Figure 6 illustrates these relationships using a Sankey diagram and reflects scientific output in relation to key concepts regarding temporality. It highlights how the study of the concept of “physical fitness” has been the most extensively developed. For the years 2022–2024, a considerable increase in the number of concepts is observed, some of which are new and related to physiology, academic success, and students. This diagram also uses color coding to illustrate the relationships between terms. It is noteworthy that the “human” variable appears in two colors but is always associated with “child” or “school child,” while “fitness” was predominant from 2017 to 2021 but was linked to “muscle strength” and “high-intensity interval training,” based on variables associated with training.

**Figure 6:**
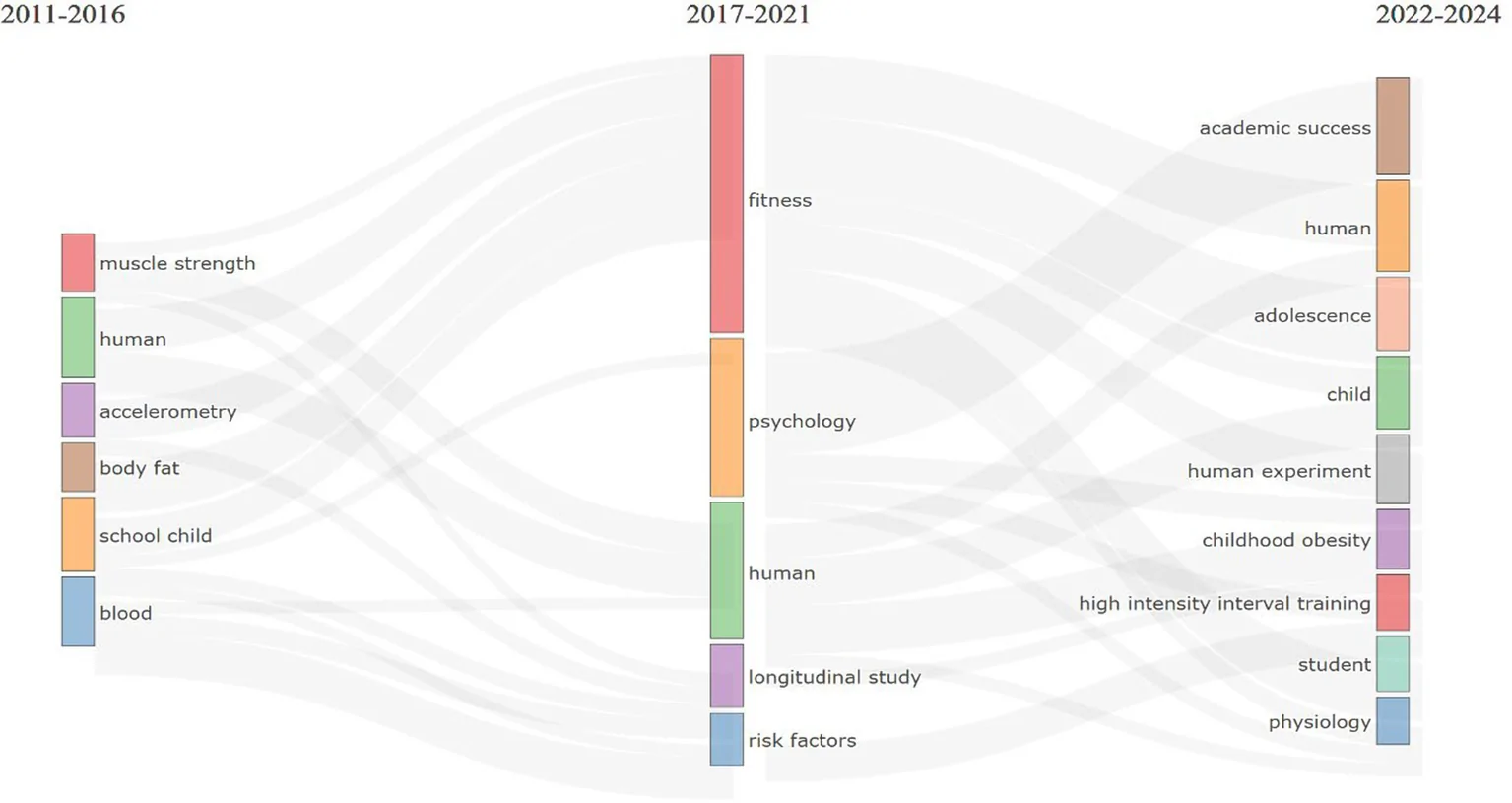
Degree of relevance and development of key concepts.

Finally, the factorial analysis reveals the trend of the concepts used (Figure 7). Thus, dimension 1 recovers 51.85% and dimension 2 recovers 23.4% of the total information. It also reveals that there are concepts that appear to be interrelated such as: (i) body mass index and sedentary behavior; (ii) school child, hand strength, and major clinical study.

**Figure 7:**
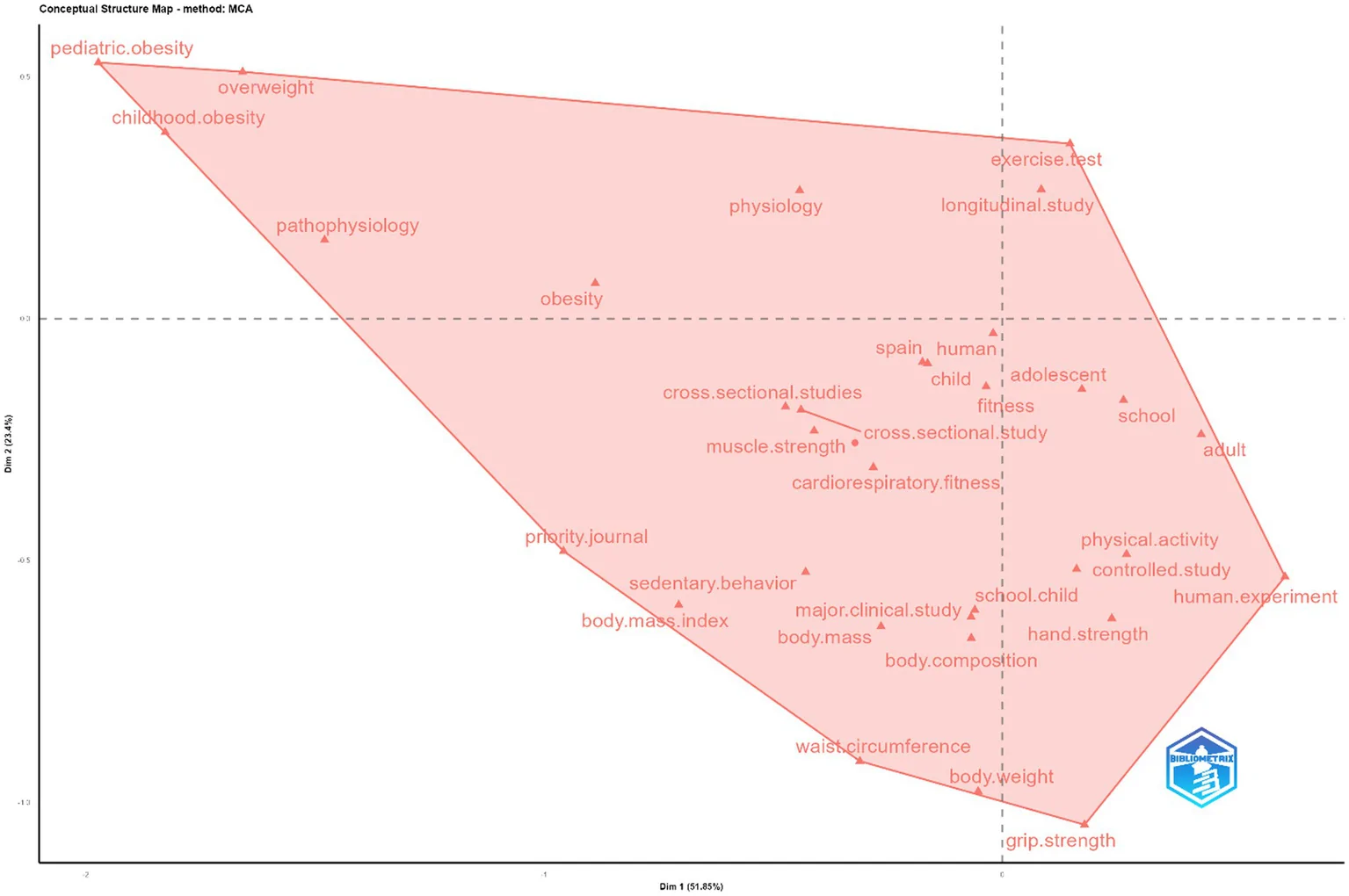
Factor analysis and dimensionality of key concepts.

### 3.5 Analysis of the authors

Table 4 shows that academic productivity reaches up to eight articles (López-Gil, J. F.), although most authors in the ranking have between three and five publications. In most cases, there is one or, at most, two publications per author. It is also worth noting that one of the most striking characteristics is that of the first author, who, with only three publications, received 989 citations, representing 37.66 % of citations among the ranked first authors in Table 4. Although both Spanish and English were considered, most articles by the 11 ranked first authors in Table 4 are written in English; one is listed as English-Spanish (Izquierdo-Gomez).

**Table 4:** Number of documents per author taking into account the first author.

Author	Number of publications	Publication language	Number of citations received
López-Gil, J. F.	8	English	130
Esteban-Cornejo, I.	7	English	446
Ortega, F. B.	5	English	280
Castro-Piñero, J.	5	English	218
Cristi-Montero, C.	5	English	15
Galan-Lopez, P.	5	English	146
Izquierdo-Gomez, R.	5	English-Spanish	70
Manzano-Carrasco, S.	5	English	91
Mora-González, J.	5	English	174
García-Hermoso, A.	4	English	67
Ruiz, J. R.	3	English	989
Total	57		2,626

Table 5 shows academic collaboration based on the number of authors per published document. The highest academic output was observed in documents with 4 to 6 authors, and the highest number of citations was observed in documents with 4–6 and ≥10 authors, which together account for 67.83% of all citations and 53.38% of documents. On the other hand, it is evident that the lowest scientific output occurs in papers with 1 to 3 authors and those with more than 10 authors, although studies conducted by more than 10 authors receive a higher total number of citations, along with those authored by 4 to 6 authors.

**Table 5:** Total number of documents by author.

Number of authors	Number of documents	Citations	Percentage of citations (%)
1–3	49	539	8.96
4–6	107	2,050	34.10
7–10	75	1,395	23.21
≥10	35	2,027	33.73
Total	266	6,011	100

The map of citations by authors is evident in Figure 8. The colors of the nodes respond to the year of publication between 2010 and 2024, along with the different connections that authors generate; likewise, the size that is assigned to each author responds to the number of publications, being the largest with the highest numbers of publications. The research considered the analysis to have a small number of authors. 2017 and 2019 are the years with the highest citations, leading Esteban-Cornejo, I., Galan-Lopez, P., García-Hermoso, A. and Artero, E.G. However, the central node focuses on 2016–2018, highlighting Ortega, F. and Castro-Piñero, J. The years with the lowest number of connections between authors are established between 2010 and 2014.

**Figure 8:**
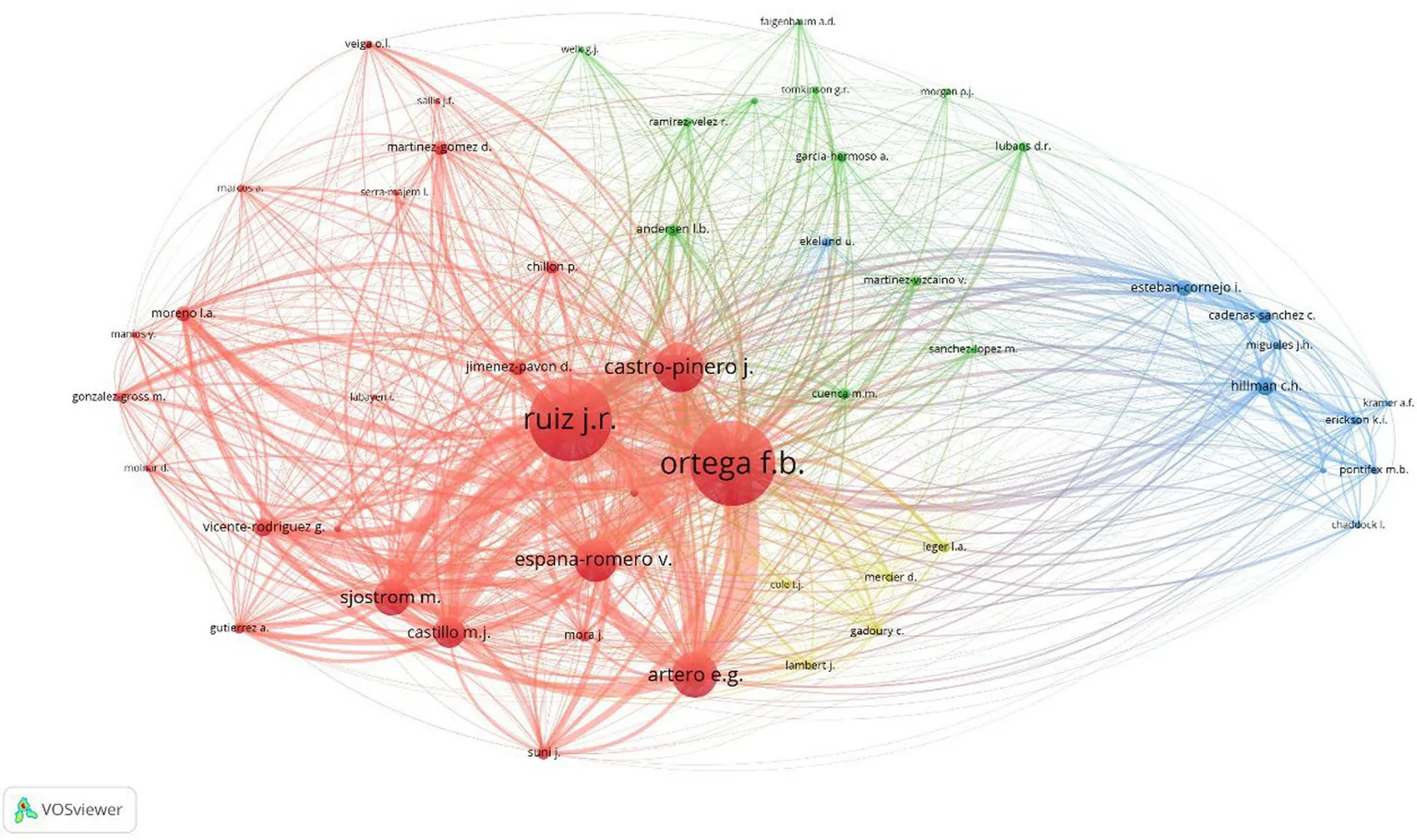
Citation by authors.

Figure 9 shows the trend of scientific production by authors between 2011 and 2024. It reveals the number of documents produced by each author in reference to the year and the number of citations received per year. The authors who stand out the most in terms of their production and citation are: Ortega, F. B., Castro-Piñero, J., and Ruiz, J. Of these three authors, the one who has maintained production for the longest time has been Castro-Piñero, J.

**Figure 9:**
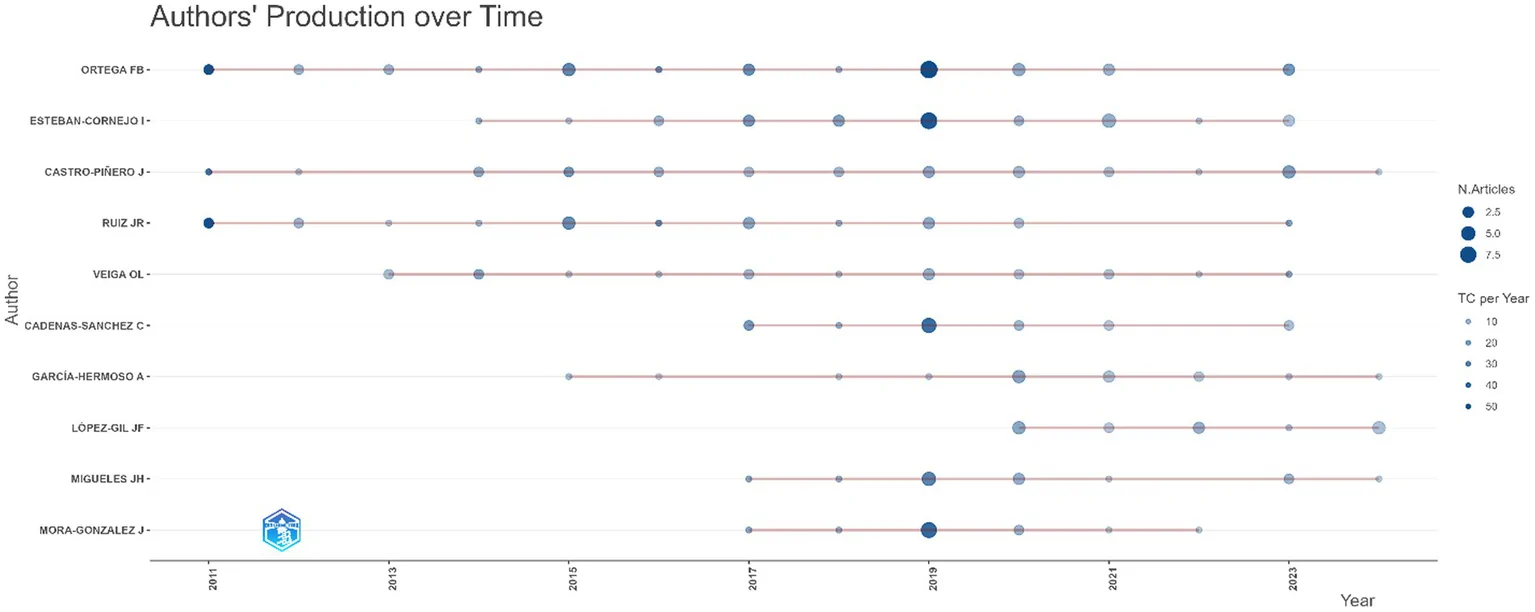
Author publication trends over time. The X-axis represents the year, and the Y-axis represents the cumulative occurrence in response to the number of papers per author.

### 3.6 Keyword analysis

Figure 10 shows the mapping of the keywords, which were considered to have at least three appearances within each investigation. The VOSviewer program identified 395 keywords, of which only 77 met the established criteria. A wide variety of terms are observed. However, those that most prevail are based on the age of the population, such as “*children*,” “*adolescents*,” and “*childhood*” between the years 2018–2019, establishing a connection with lifestyle factors such as *“health,” “habits,” “exercise,” “school”* and physiological factors including “*obesity*,” “*adiposity*,” “*body mass index*” and “*cardiovascular fitness*” between the years 2018–2020. The year with the lowest number of keywords is 2021; on the contrary, 2018 and 2019 are where the most large-scale nodes are observed.

**Figure 10:**
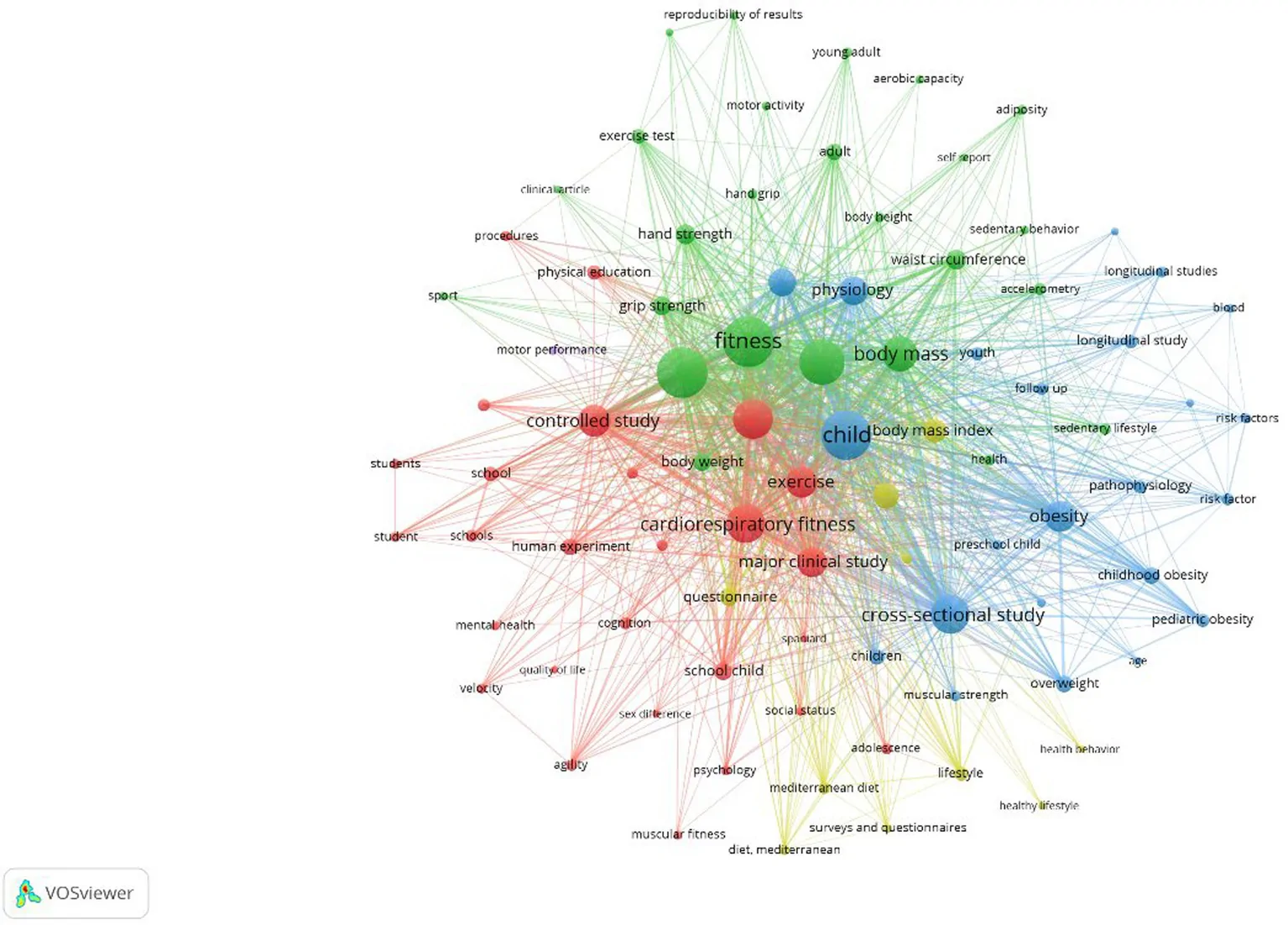
Occurrence by keywords between 2017 and 2021.

### 3.7 Analysis of journals

Figure 11 presents the 10 most relevant journals that stand out for their H-index. This can help determine bibliometric indicators that indicate the h number of publications of an author or a journal that have received an h number of citations, highlighting the dissemination of research. Among the journals with the highest H-index values are the “International Journal of Environmental Research and Public Health” and the “Scandinavian Journal of Medicine and Science in Sports”, while among those with the lowest H-index values are the “British Journal of Sports Medicine” and “BMC Public Health”.

**Figure 11:**
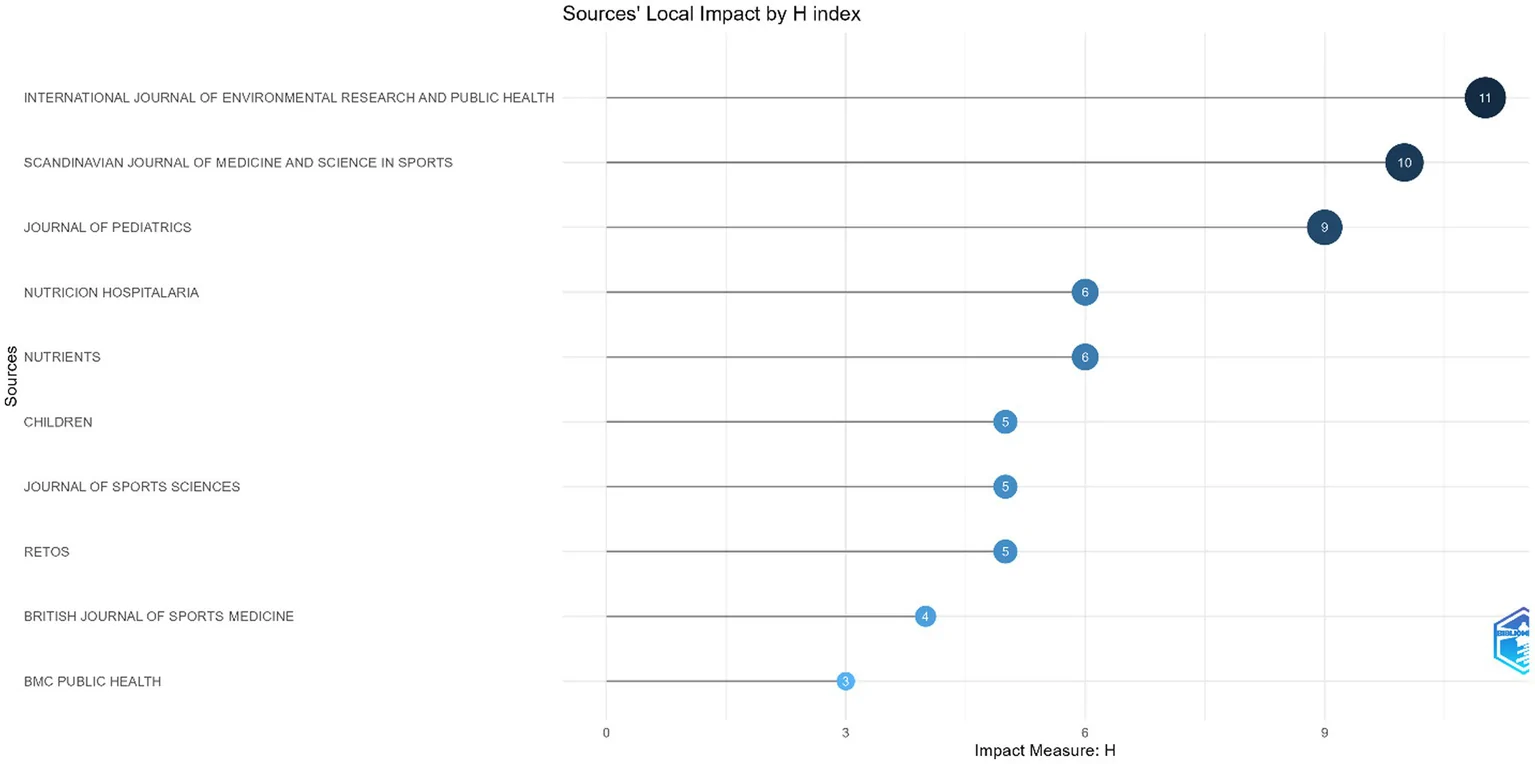
H-index of main journals.

Another important bibliometric indicator lies in understanding the scientific production of journals in response to the year. Thus, it is detailed that among the top five journals, International Journal of Environmental Research and Public Health and Scandinavian Journal of Medicine and Science in Sports stand out with about 20 studies published between 2022 and 2024 (Figure 12).

**Figure 12:**
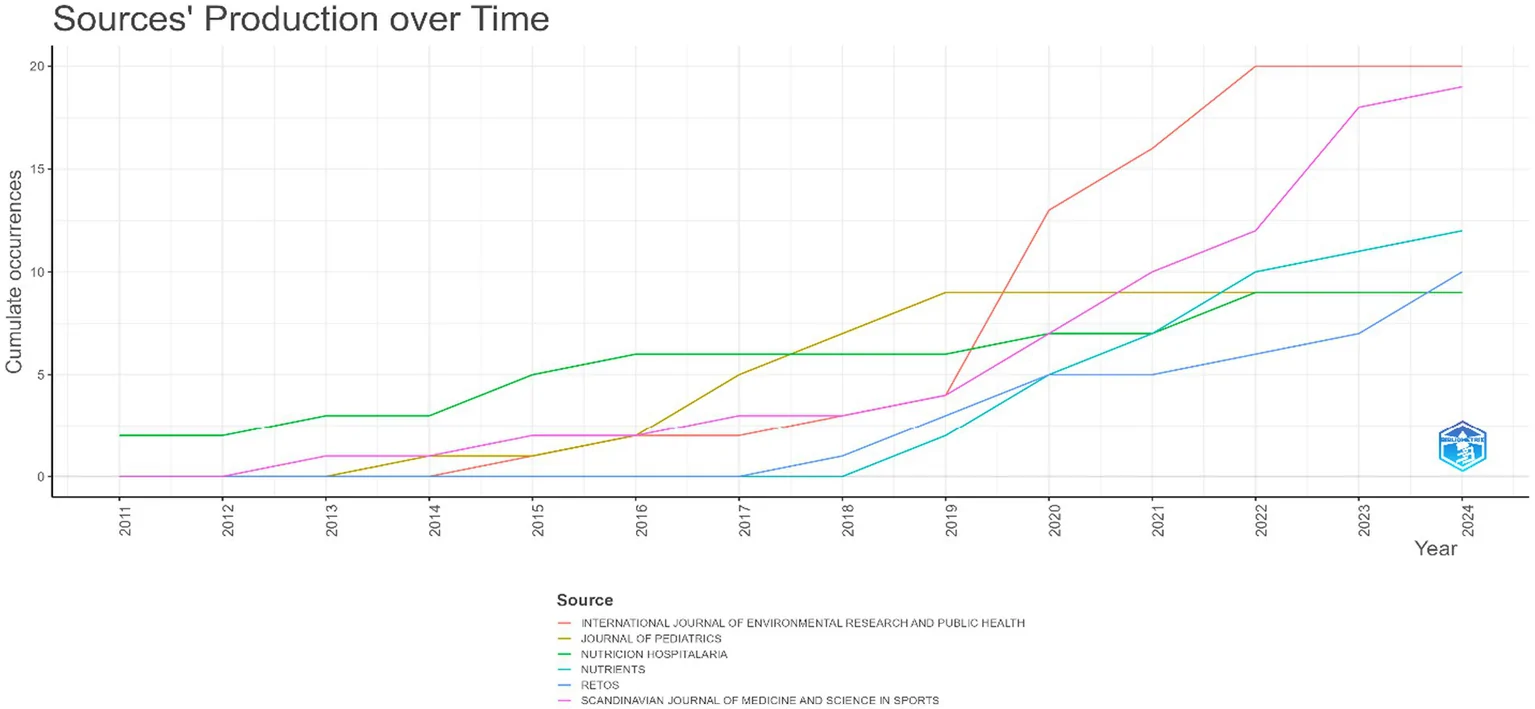
Annual scientific production of the main journals.

In addition to the above, the mapping of journals by co-citations found 1,066 documents in total. The minimum value of citations is outside of 20 per journal, and, therefore, 32 meet the criteria. The journals with the most significant impact were: Brit J Sport Med (*n* = 166), Int Journal Obesity (*n* = 101), J Sport Sci (*n* = 84), and Med Sci Sport Exer (*n* = 82) (see Figure 13).

**Figure 13:**
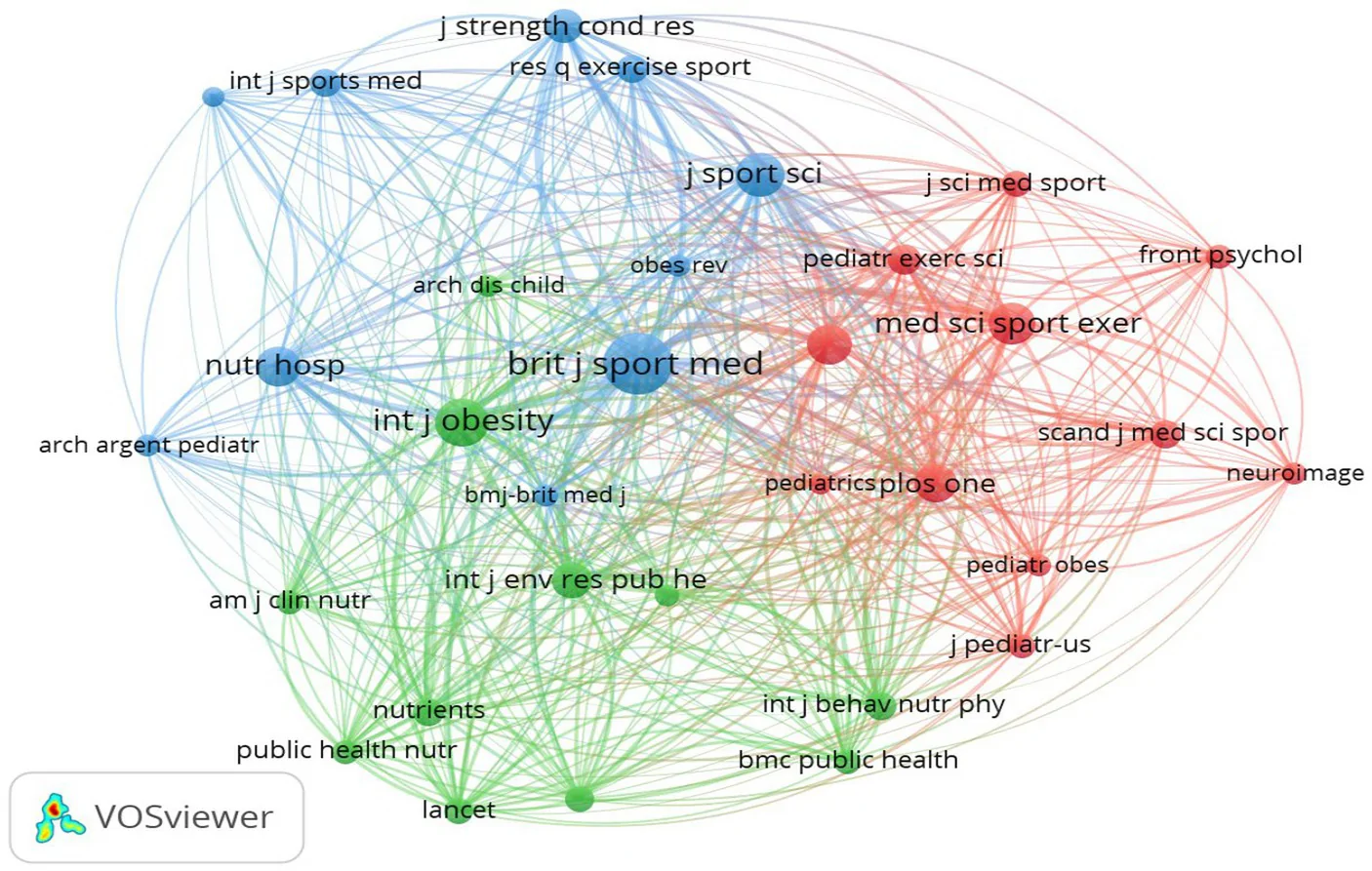
Co-citation by journal.

### 3.8 Analysis by country

The countries that establish citation collaboration relationships are shown in Figure 14. In total, 32 countries have been found to have participated in the research subject, of which 22 countries published more than two documents, and only eight countries have published more than five studies. The countries with the highest levels of publication are: Spain (*n* = 63) documents and (*n* = 2,130) citations; Sweden (*n* = 14) documents and (*n* = 1,469) citations; Chile with (*n* = 13) documents and (n = 91) citations; United States (*n* = 12) documents and (*n* = 687) citations; Colombia with (n = 6) documents and (*n* = 56) citations and Finland (n = 3) documents and (*n* = 677) citations.

**Figure 14:**
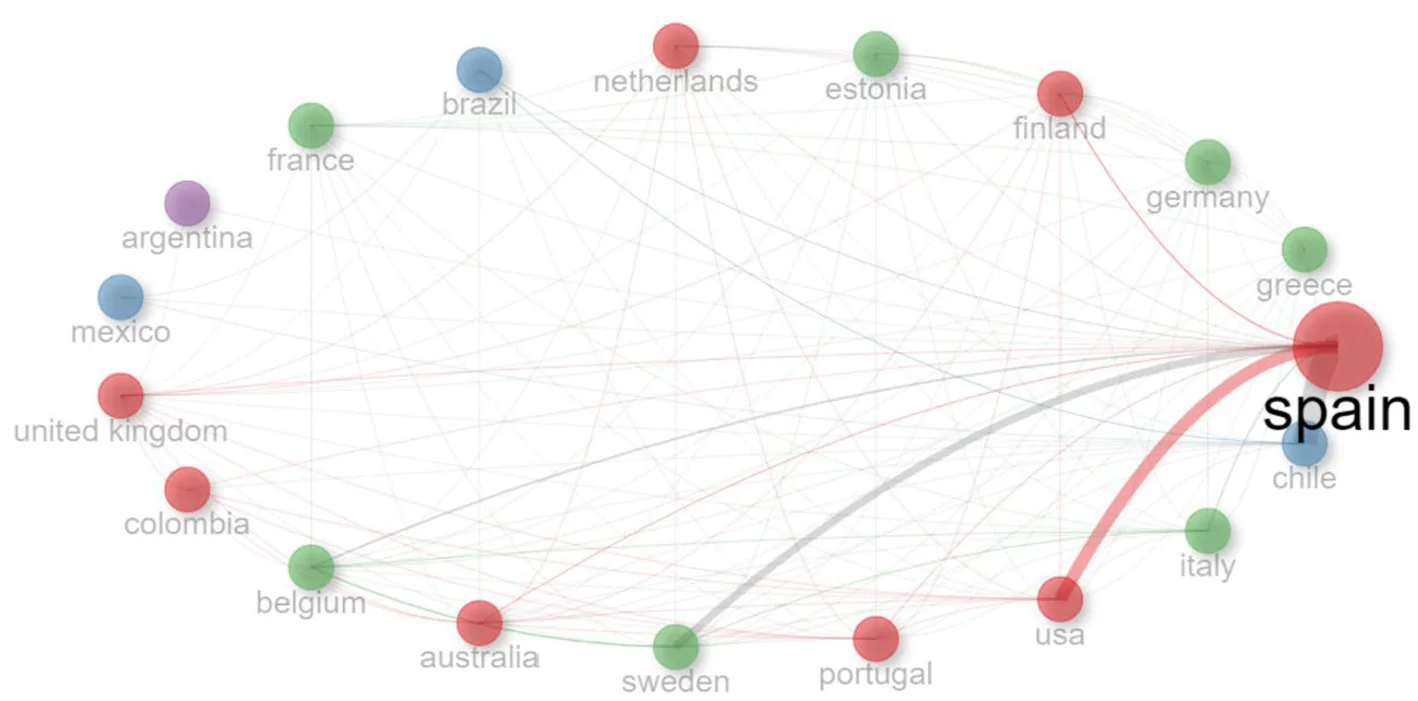
Number of countries producing knowledge related to the ALPHA-Fitness battery.

To establish academic cooperation between countries, the production of a single country and the production among several countries are determined. Of the top 10 countries, none of them has prioritized the study of a single country. This information demonstrates the importance of this topic for the scientific community. It is noteworthy how the country that produces the most knowledge (Spain) has an equal proportion between multiple production and scientific production from the same country (see Figure 15).

**Figure 15:**
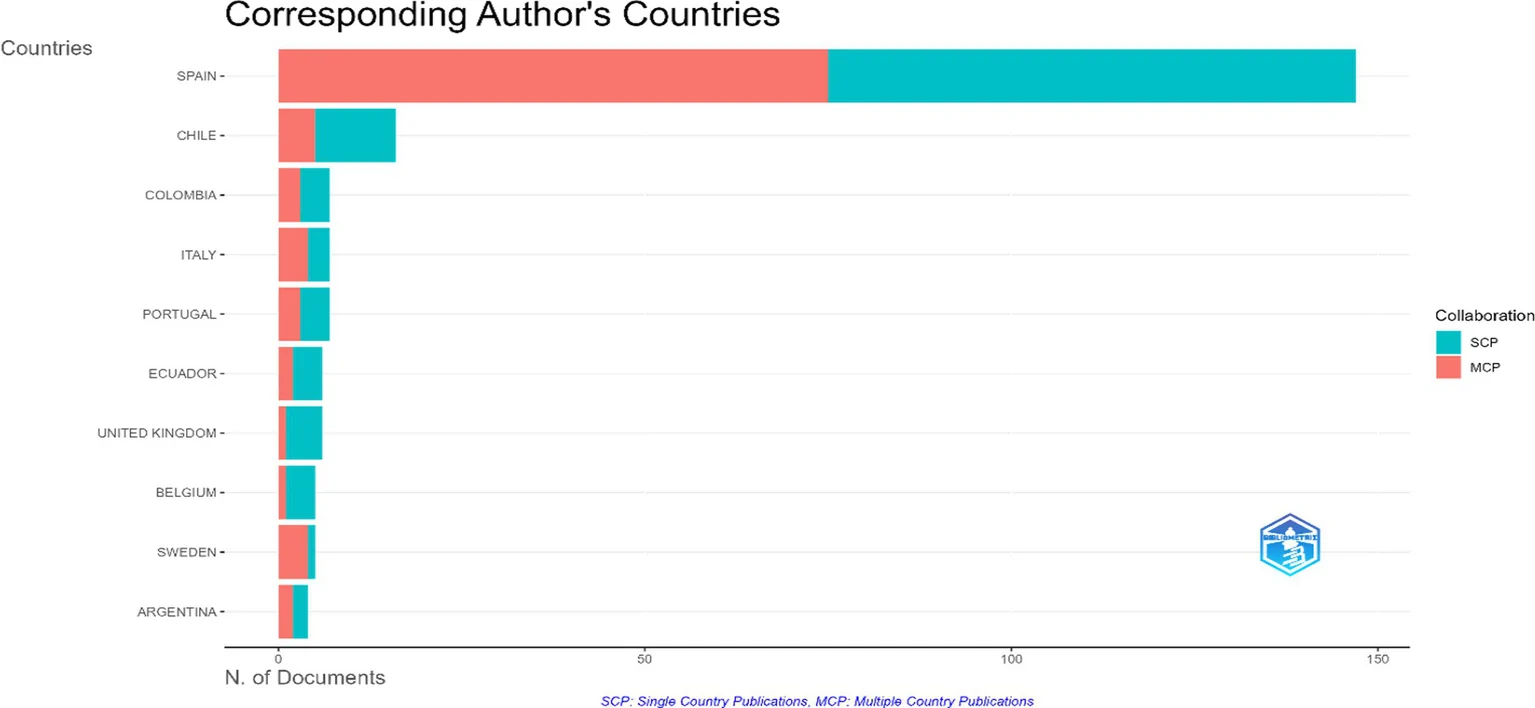
Scientific production by number of countries.

### 3.9 Organizations

For co-authorship by institutional affiliation, this was quantified based on the number of documents linked to each organization through at least one co-author, using VOSviewer’s co-authorship network. Since a single document may be linked to more than one organization, Figure 16 should be interpreted with caution, as it does not represent an absolute share of the total corpus (*n* = 266). Among the top 10 organizations that have contributed to the study of scientific production, the University of Granada stands out with 189 documents linked through this network, while the ninth and tenth organizations have only contributed 13.75 and 13.22%, respectively, relative to the top-ranked organization (see Figure 16).

**Figure 16:**
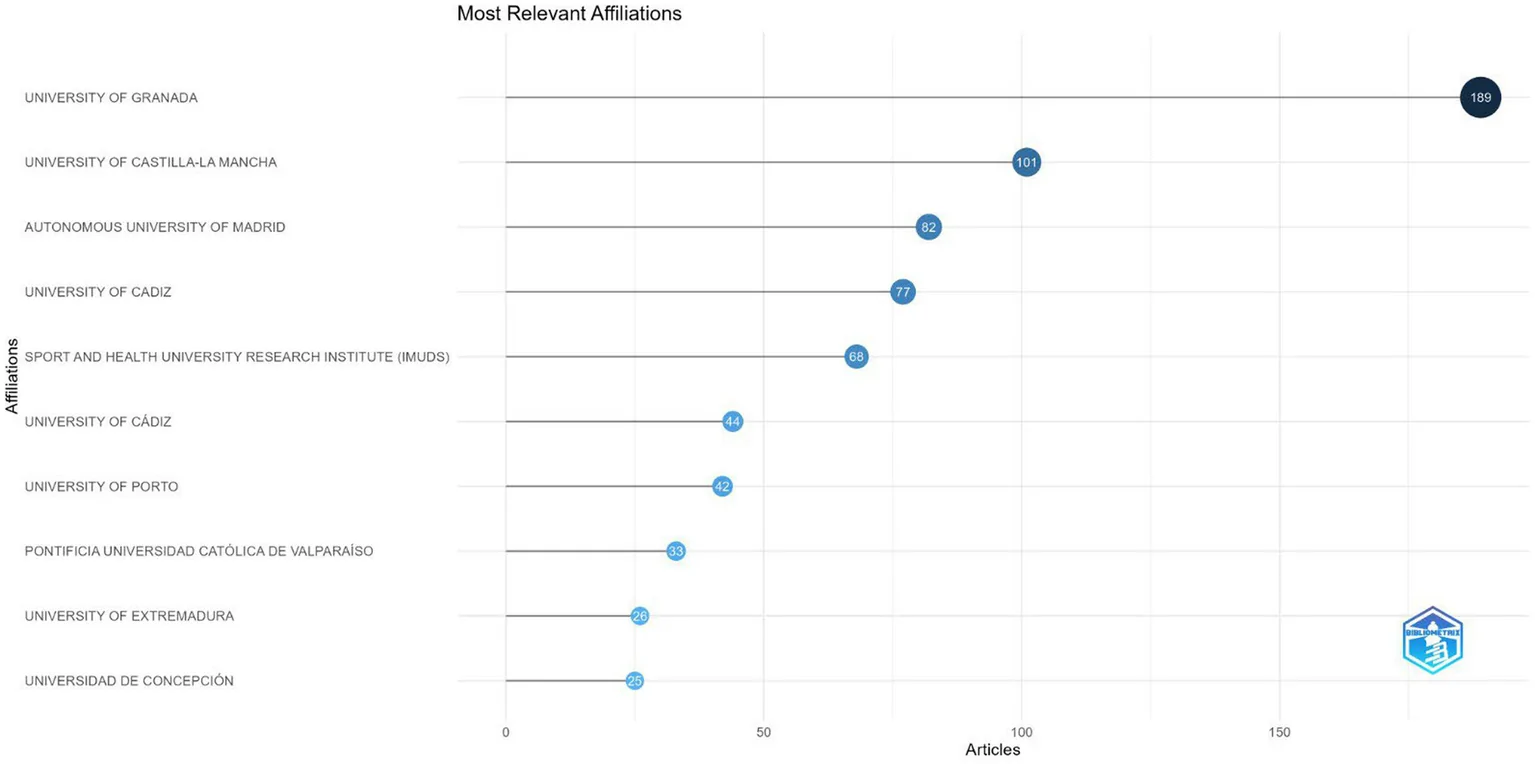
Co-authorship by organizations.

## 4 Discussion

As far as we know, this is the first review to conduct a bibliometric analysis of scientific publications related to the use of the ALPHA Fitness battery for assessing physical fitness in children and adolescents. Analyzing the evolution and trends in the number of studies related to the ALPHA-Fitness battery over the past 15 years has revealed several key insights. 266 documents related to the study of the ALPHA-Fitness battery have been published between 2010 and February 28, 2025. The most significant production has been generated between 2019 and 2024, representing 69.2% of total scientific production. There is a preference for the development of research articles (98.87%) with three review articles identified (see Table 2). The years 2019 and 2017 are the ones that receive the most citations. There are at least 10 documents that have received 50 citations. From 2010 through February 2025, three review articles were identified in the corpus; no prior standalone bibliometric mapping of the ALPHA-Fitness battery had been published before the present study. The average number of citations per article was 22.60. Articles with 4 to 10 authors accounted for 68.42% of all documents and 57.31% of all citations. The authors with the most significant number of documents, taking into consideration the first author, are López-Gil, J. F. (*n* = 8), Esteban-Cornejo, I (*n* = 7), Ortega, F. B. (*n* = 5), Castro-Piñero, J. (*n* = 5) and Cristi-Montero, C. (*n* = 5).

The most recent concepts are associated with evaluation processes in school environments and associated with cognitive and academic performance. The journals that have published the most significant number of documents and received the most citations are the British Journal of Sports Medicine, the International Journal of Environmental Research and Public Health. MDPI (Multidisciplinary Digital Publishing Institute) is the publishing group that has published the most significant number of documents, and BMJ Publishing Group has received the highest number of citations. According to Web of Science (WoS) Core, the most developed areas are Sport Sciences, Nutrition & Dietetics, Public, Environmental & Occupational Health, Environmental Sciences, and Pediatrics. The study on the ALPHA-Fitness battery has been published in English and Spanish, with English being the predominant. One hundred sixty-three organizations have produced knowledge about the ALPHA-Fitness battery, highlighting Spanish universities such as the University of Granada.

Despite the growing number of documents aimed at evaluating PF and PA levels in children and adolescents (34–36), as well as the significant increase in studies that use different batteries and tests to measure and quantify certain physical qualities (37, 38), the number of bibliometric review studies that analyze the trends of scientific production on the use of these batteries in children and adolescents is still scarce. Likewise, despite the investigative interest evidenced by the documents published annually on the use of the ALPHA-Fitness battery (*R*^2^ = 0.9242 for the annual publication trend; Figure 2), to date, no prior standalone bibliometric mapping of the ALPHA-Fitness battery had applied classical bibliometric laws to contrast the results found in this study.

It has been determined that Spain was the country/region that made the most publications on the study of the ALPHA-Fitness battery. Spanish-speaking countries such as Chile, Ecuador, Argentina, and Colombia were also included. These findings are related to other studies on psychomotricity in childhood (23). However, another study that analyzed the scientific evolution of PA in preschool children reveals that the countries with the highest scientific production are not Spanish speaking, highlighting the United States, Australia, China, the United Kingdom, and Canada (39). Another bibliometric study that studies the impact of the built environment on PA in children and adolescents has revealed how 47 countries have contributed to this field of knowledge, led by the US, Canada, Australia, England, and China. These results point out the multi-territorial interest carried out with different research initiatives that awaken the study of PF and the evaluation of PA in the school-age population (40).

From this perspective, the study developed by Marques et al. (15) reveals that there are 25 batteries to monitor the health status of children and adolescents, where the academic efforts of European, Asian, American, and Oceanian countries are evidenced. This is how the knowledge that is produced about the different validated and standardized PF batteries allows comparisons and monitoring with specific population groups and, on the other hand, allows comparison with other contexts, ages, and geographical locations with low costs (41). This scenario reflects, in turn, that PF is a multi-component construct that cannot be understood from the evaluation of some tests, so it is necessary to understand that batteries seek to integrate the associations that occur between PF constructs and health biomarkers (1, 42).

The present review study has identified that the use of the ALPHA-Fitness battery pursues different objectives associated with health, among them the study developed by López-Gil et al. (43) that aimed to describe and analyze the level of PF and PA in schoolchildren to determine adherence to the Mediterranean diet (MedDiet), a case similar to other studies developed by Galan-Lopez et al. (44, 45) that sought to establish the relationship between PF, body composition and adherence to MedDiet, concluding that adherence to MedDiet is related to high levels of resistance, where girls achieve higher health-related PF levels than boys. Another study that analyzed body weight with PF and blood pressure (BP) for health concluded that children with average weight obtained better PF and BP results than those overweight children. However, no relationship was determined with sedentary behaviors (46). An investigation developed by Quiñonero Martínez et al. (47) has highlighted that sedentary habits, obesity levels, and low PA levels are worrying, stating that, although in the school context, the initiatives that are developed can favor the reduction of these sedentary behaviors, the teaching time that is dedicated in the school environment to generate a physically active education is not enough to improve PF levels. To this end, different public policies are needed to help counteract the harmful effects that can be triggered by a sedentary lifestyle and physical inactivity, as well as other non-communicable diseases (48, 49).

Research development is also equally important, as well as academic cooperation between different authors, organizations, and countries, seeking to generate concrete lines of action to minimize the health problems associated with a sedentary lifestyle (50), the time of exposure in front of screens, the low levels of PF and PA in children and adolescents and the promotion of healthy lifestyles (51). Mapping the knowledge that has been generated on a battery that seeks to measure the levels of PF allows, in turn, to analyze the trends and the objectives that are sought with the evaluation of the different components of the PF and, thus, understand the gaps of knowledge and the unexplored lines of research. Given this, it has been confirmed that by deepening a topic, the research horizons can be expanded, revealing that there are emerging concepts associated with the use of the ALPHA-Fitness battery that, in turn, project future lines of research. Among the most relevant concepts are: “*Fat*,” “*Students*,” “*Brain*,” “*Academic-Performance*,” “*School-Aged Children*,” and “*Cognitive Function*.” These findings reinforce the importance of health education in the school context, seeking to generate processes of awareness and the creation of habits and active lifestyles (52) to combat sedentary behaviors (53), obesity (54), and physical inactivity (55).

To this end, it is suggested that different review studies be continued to allow us to relate the various variables associated with the health of the population. In the same way, the main contribution of this study lies in understanding the research trends, as well as the production of knowledge generated on the use of the ALPHA-Fitness battery about the occurrence of the most studied concepts by the scientific literature, as well as the academic efforts that different authors, institutional affiliations and countries have been developing. All this is because studies of bibliometric nature generalize the knowledge of a particular topic for emerging and experienced researchers interested in continuing to delve into a specific topic that needs more significant research development (20, 21).

This bibliometric review identified similar trends in scientific output. The period 2019–2024 was the most productive (*n* = 184 publications), and 2019 also recorded the highest number of citations (*n* = 807), followed by 2017 (*n* = 767). These findings are consistent with other bibliometric analyses reporting an average of 44 publications per year (56). Likewise, high citation counts were observed in earlier years with very few publications, such as 2011 (3 publications; 747 citations). Marked fluctuations were also evident between 2014 and 2019 (e.g., 471 to 807 citations). This suggests that, although older documents tend to accumulate more citations over time, this pattern is not consistent throughout the entire period, as other early years with similarly low output (e.g., 2012, 2 publications) recorded comparatively low citation counts (119 citations). This partially aligns with prior studies addressing a similar subject matter (57), which report that citation accumulation tends to favor older documents, although the pattern was not uniform or generalizable. It should be noted that most publications were in English, with only two identified as bilingual (English-Spanish) in the present sample.

Finally, the stored data pose challenges for the scientific community, given that the literature focuses primarily on Spain, where it accounts for nearly 23.7% of ALPHA-Fitness battery total output and 35.4% of all citations; however, the lack of PA is currently considered a public health concern (58), where systematic reviews involving more than 43,000 children have concluded that minimum levels of PA are not being met (59). Therefore, this bibliometric review demonstrates that ALPHA-Fitness battery must remain an essential tool for assessing the health status of populations, and interventions are needed in other countries and on other continents to strengthen these findings on a global scale.

The 10 articles with the highest number of citations were identified, with the article by Ruiz et al. having the highest number of citations (*n* = 543), while the most cited review study is that of Ruiz et al. (33) (*n* = 253). Research articles have received 1,352 citations, while review studies total *n* = 532 citations (see Table 6).

**Table 6:** Most cited articles.

Author and PY	TD	Journal	Sample and cont	Aged (Yrs)	Var	Main results	Conclusions	Tot cit
Ruiz et al. (17)	Art	British Journal of Sports Medicine	11 methodological studies, 3 systematic reviews, and cross-sectional studies.	-	PF and Health	The battery of physical fitness in children and adolescents in the school environment proved to be safe from analysis in cross-sectional studies, three systematic reviews and 11 methodological studies to examine the 5 tests from existing literature.	The implementation times of the ALPHA-Fitness Battery last approximately 2 h for a group of 20 students, where its implementation is valid, reliable, feasible and safe for the control of the physical health of students.	543
Ruiz et al. (33)	Rev	British Journal of Sports Medicine	7 studies (9,280 children).	8–19	Cardiorespiratory fitness, cardiovascular disease risk	Cardiovascular disease risk was already present in boys (6–39%) and girls (6–86%). Boys with low fitness (<41.8 mL/kg/min) had a 5.7 times greater likelihood of having cardiovascular disease risk (95% CI 4.8 to 6.7). The comparable diagnostic OR for girls with low fitness (<34.6 mL/kg/min) was 3.6 (95% CI 3.0 to 4.3). The 95% confidence region of cardiorespiratory fitness associated with low cardiovascular disease risk ranges, 41.8–47.0 mL/kg/min in boys (eg, stages 6–8 for a boy aged 15 years) and 34.6–39.5 mL/kg/min in girls (eg, stages 3–5 for a girl aged 15 years).	Fitness levels below 42 and 35 mL/kg/min for boys and girls, respectively, should raise a red flag. These translate to 6 and 3 stages on the shuttle run test for a boy and a girl, both aged 15 years, respectively. These cut points identify children and adolescents who may benefit from primary and secondary cardiovascular prevention programming.	253
Ruiz et al. (16)	Art	Nutrición Hospitalaria	-	-	PF and health	The ALPHA-Fitness battery was created by establishing four phases: 1. literature review; 2. reliability and validity studies; 3. analysis of prerequisites; 4. proposal of the battery for evaluating physical condition in children and adolescents.	A reliable and valid instrument for assessing physical condition in schoolchildren, with three variants: evidence-based, high-priority, and extended ALPHA-Fitness.	193
Ortega et al. (60)	Rev	Sports Medicine	22 studies	4–6	20 m shuttle-run, handgrip-strength, standing long-jump, 4 × 10 m shuttle run, one-leg-stance	Most studies focused on reliability of the fitness tests (*n* = 21; 96%), while very few focused on validity (0 criterion-related validity and 4 (18%) convergent validity) or relationship with health outcomes (0 longitudinal and 1 (5%) cross-sectional study). Motor fitness, particularly balance, was the most studied fitness component, while cardiorespiratory fitness was the least studied. After analyzing the information retrieved in the current systematic review about fitness testing in preschool children, we propose the PREFIT battery, field-based FITness testing in PREschool children.	The present systematic review has identified a need for further research on the validity of fitness tests in preschool children, as well as on their relationship with health.	191
De Miguel-Etayo et al. (61)	Art	International Journal of Obesity	10,302 children	6–10.9	Flamingo balance test, flexibility, handgrip strength, standing long jump, 40-m sprint, 20-m shuttle run	Boys obtained better results than girls in speed, lower and upper limb strength, and cardiorespiratory capacity, while girls obtained better results in balance and flexibility. Older children performed better than younger ones, except in boys’ cardiorespiratory capacity and girls’ flexibility.	The results provide sex- and age-specific physical fitness reference standards for European children.	159
Muros et al. (62)	Art	Nutrition	515 children	10.6 ± 0.5	PF, PA, BMI, skin folds, waist circumference, self-concept	Adherence to a MedDiet was consistently and negatively associated with body fat percentage (*r* = −0.302) and subscapular skinfold thickness (*r* = −0.329). Positive associations were found with PAQ-C (*r* = 0.277), self-esteem (*r* = 0.301) and self-concept (*r* = 0.234), and with physical fitness, especially with explosive leg power (*r* = 0.355). Positive correlations were found with handgrip strength in boys (*r* = 0.323), while negative correlations were found with screen time in girls (*r* = −0.511).	The inverse associations between body composition parameters and KIDMED scores suggest that healthy and overweight children are more likely to have high adherence than obese children. Adherence to a MedDiet may be related to mental well-being through aspects such as self-esteem and self-concept.	126
Esteban-Cornejo et al. (63)	Art	Neuroimage	101 children	8–11	PF, gray matter tissue, academic performance, total brain volume, sex, BMI, parental education	Higher cardiorespiratory fitness was related to greater gray matter volumes (*p* < 0.001, k = 64) in 7 clusters with *β* ranging from 0.493 to 0.575; specifically in frontal regions (i.e., premotor cortex and supplementary motor cortex), subcortical regions (i.e., hippocampus and caudate), temporal regions (i.e., inferior temporal gyrus and parahippocampal gyrus), and calcarine cortex. Higher speed-agility was associated with greater gray matter volumes (p < 0.001, k = 57) in 2 clusters (i.e., inferior frontal gyrus and superior temporal gyrus) with β ranging from 0.564 to 0.611. Muscular fitness was not independently associated with greater gray matter volume in any brain region.	Cardiorespiratory fitness and speed-agility, but not muscular fitness, may be independently associated with greater volume of numerous cortical and subcortical brain structures; furthermore, some of these brain structures may be related to better academic performance. Importantly, the identified associations of fitness and gray matter volume were different for each fitness component.	124
Esteban-Cornejo et al. (64)	Art	The Journal of Pediatrics	2,038 youths	6–18	Muscular strength, Handgrip strength, standing long jump	Cardiorespiratory capacity and motor ability were independently associated with all academic variables in youth, even after adjustment for fitness and fatness indicators (all *p* ≤ 0.001), whereas muscular strength was not associated with academic performance independent of the other 2 physical fitness components. In addition, the combined adverse effects of low cardiorespiratory capacity and motor ability on academic performance were observed across the risk groups (p for trend < 0.001).	Young people who had lower levels of both cardiorespiratory fitness and motor ability had poorer grades in academic subjects. Thus, high levels of cardiorespiratory and motor fitness may reduce to some extent the risk of school failure, a possibility that deserves to be evaluated in intervention trials.	106
Ramírez-Vélez et al. (65)	Art	PloS One	229	9–17.9	BMI, Waist circumference, triceps and subscapular skinfold, body fat (%), handgrip and standing long jump, speed/agility (4 × 10 m), flexibility, 20 m shuttle run	For the musculoskeletal, motor, flexibility, and cardiorespiratory components, we found adequate reliability patterns in terms of systematic errors (bias) and random error (95% limits of agreement). When physical fitness assessments were performed twice, the systematic error was close to 0 for all tests, except for sit-and-reach (mean difference: −1.03% [95% CI = −4.35% to −2.28%]).	The “Fuprecol Study” health-related physical fitness battery, administered by physical education teachers, was reliable for measuring the health-related components of physical fitness in children and adolescents aged 9 to 17.9 years in a school setting in Colombia.	101
Bianco et al. (66)	Rev	International Journal of Occupational Medicine and Environmental Health	100 manuscripts		20 m shuttle run test, handgrip strength, standing broad jump, sit-up test, 4 × 10 m	These fitness tests have been finally selected and incorporated into the Adolescents and Surveillance System for the Obesity prevention - Fitness Test Battery (ASSO-FTB) and will be adopted within the ASSO Project for evaluation purposes. This instrument could be also provided to teachers and people working in schools in order to assess the physical fitness of adolescents over time and prevent obesity and related diseases.	The ASSO Project has been created with the intention of providing teachers and people working in schools within a fitness context with an instrument able to monitor physical fitness over time in order to prevent obesity and future related diseases. The ultimate aim of this paper is to offer an accessible, low cost battery of fitness tests that could be applied worldwide within a school environment.	88

The average number of citations per article was calculated from the date of publication until February 28, 2025*. PY, publication year; TD, type document; Con, context; Yrs, years; Var, variables; Art, article; Rev, review; BMI, body mass index; m, meters; PREFIT, field-based FITness testing in PREschool children; PA, physical activity; PF, physical fitness; CRF, cardiorespiratory fitness; ASSO-FTB, Adolescents and Surveillance System for the Obesity prevention – Fitness Test Battery; PAQ-C: Physical Activity Questionnaire; KIDMED: Índice KIDMED.

### 4.1 Limitations

This study has several limitations related to the heterogeneity of the study populations and the characteristics evaluated, as well as to the various names used for the ALPHA-Fitness battery, since it was found that three different versions were used in the various studies identified. This poses a challenge when generalizing the results. Furthermore, this study was limited to documents indexed in only a few databases, such as WoS, Scopus, and PubMed, which could introduce language-related biases. Although these are widely used databases for conducting bibliometric studies, it is suggested that other databases be explored in future studies. Another limitation is associated with the absence of an analysis of protocol standardization or the specific characteristics of the populations in which the battery was used. For all these reasons, these limitations must be taken into account when generalizing the findings. This is because the present study sought to represent the landscape of scientific evidence reflected in the indexed literature.

## 5 Conclusion

This study has demonstrated that the ALPHA-Fitness battery has become established in schools as a tool for analyzing and assessing the physical fitness of children and adolescents. Upon analyzing the various articles, the most widely used type of battery was the extended test, which allows for the assessment of physical fitness in young people and is well-documented in the literature. However, the research is largely focused on Spain, where the leading authors are based, making it necessary to continue investigating the ALPHA-Fitness battery in other contexts. In the analysis of the selected information, it is evident that researchers’ interest is centered on empirical articles, while review studies are less prevalent; therefore, this bibliometric analysis allows for the identification of conclusive reviews and their key findings, thereby enabling the scientific community to compile information into a single document. When examining the most important words and terms in physical fitness testing, the following stand out: “children,” “adolescents,” “cardiorespiratory fitness,” “obesity,” “childhood,” “health,” “physical activity,” “reliability,” and “youth.” Scientific research has focused on students’ physical fitness, cardiorespiratory capacity, and disease prediction aspects that are analyzed and evaluated using readily available tools, which also help Sports Science teachers make the most of their time in the school setting. There is a particular global interest in students’ health and physical fitness, an area in which teachers can apply reliable test batteries in sports practice; it has been observed that the battery does not necessarily have to be implemented in its entirety, but rather that modifications can be made to it.

## Data Availability

The original contributions presented in the study are included in the article, further inquiries can be directed to the corresponding author.
